# An Optimized Bioassay for Screening Combined Anticoronaviral Compounds for Efficacy against Feline Infectious Peritonitis Virus with Pharmacokinetic Analyses of GS-441524, Remdesivir, and Molnupiravir in Cats

**DOI:** 10.3390/v14112429

**Published:** 2022-11-01

**Authors:** Sarah Cook, Luke Wittenburg, Victoria C. Yan, Jacob H. Theil, Diego Castillo, Krystle L. Reagan, Sonyia Williams, Cong-Dat Pham, Chun Li, Florian L. Muller, Brian G. Murphy

**Affiliations:** 1 Department of Pathology, Microbiology, and Immunology, School of Veterinary Medicine, University of California-Davis, Davis, CA 95616, USA; ldcastillo@ucdavis.edu (D.C.); sywilliams@ucdavis.edu (S.W.); bmurphy@ucdavis.edu (B.G.M.); 2 Department of Surgical and Radiological Sciences, School of Veterinary Medicine, University of California-Davis, Davis, CA 95616, USA; lwittenburg@ucdavis.edu; 3 Department of Cancer Systems Imaging, University of Texas MD Anderson Cancer Center, Houston, TX 77054, USA; yan22v@mtholyoke.edu (V.C.Y.); cpham3@mdanderson.org (C.-D.P.); cli@mdanderson.org (C.L.); 4 Office of Research, Campus Veterinary Services, University of California-Davis, Davis, CA 95616, USA; jhtheil@ucdavis.edu; 5 Department of Veterinary Medicine and Epidemiology, School of Veterinary Medicine, University of California-Davis, Davis, CA 95616, USA; kreagan@ucdavis.edu; 6 Sporos Bioventures, @JLABS Suite 201, 2450 Holcombe Blvd, Houston, TX 77021, USA; aettius@aol.com

**Keywords:** feline infectious peritonitis, FIPV, coronavirus, antiviral, pharmacokinetics, combined anti-coronaviral therapy

## Abstract

Feline infectious peritonitis (FIP) is a fatal disease of cats that currently lacks licensed and affordable vaccines or antiviral therapeutics. The disease has a spectrum of clinical presentations including an effusive (“wet”) form and non-effusive (“dry”) form, both of which may be complicated by neurologic or ocular involvement. The feline coronavirus (FCoV) biotype, termed feline infectious peritonitis virus (FIPV), is the etiologic agent of FIP. The objective of this study was to determine and compare the in vitro antiviral efficacies of the viral protease inhibitors GC376 and nirmatrelvir and the nucleoside analogs remdesivir (RDV), GS-441524, molnupiravir (MPV; EIDD-2801), and β-D-N^4^-hydroxycytidine (NHC; EIDD-1931). These antiviral agents were functionally evaluated using an optimized in vitro bioassay system. Antivirals were assessed as monotherapies against FIPV serotypes I and II and as combined anticoronaviral therapies (CACT) against FIPV serotype II, which provided evidence for synergy for selected combinations. We also determined the pharmacokinetic properties of MPV, GS-441524, and RDV after oral administration to cats in vivo as well as after intravenous administration of RDV. We established that orally administered MPV at 10 mg/kg, GS-441524 and RDV at 25 mg/kg, and intravenously administered RDV at 7 mg/kg achieves plasma levels greater than the established corresponding EC_50_ values, which are sustained over 24 h for GS-441514 and RDV.

## 1. Introduction

Feline infectious peritonitis (FIP) is a common and generally fatal viral disease of domestic cats caused by various genetic mutants of feline coronavirus (FCoV), collectively referred to as FIP virus (FIPV). Although the complex pathogenesis of FIP remains incompletely understood and involves both pathogen and host factors, FIP disease is thought to result from de novo mutations in the relatively benign coronavirus biotype, feline enteric coronavirus (FECV) [1,2]. A critical switch in viral tropism from intestinal enterocytes to monocytes/macrophages, facilitating systemic spread and inflammation [1,2], results from these viral mutations. FCoV is also categorized into serotypes I and II, with serotype II evolving as a result of a historic recombination event between FCoV and canine coronavirus (CCoV). This recombination resulted in the stable incorporation of the CCoV *spike* gene within the FCoV genome [3]. The *spike* gene encodes an exposed coronaviral surface protein and serves as a target of antibody production by the infected host [4]. Although FCoVs are currently classified as a single viral species within the *Alphacoronavirus 1* species, given the biological significance of the coronavirus spike protein in host cell tropism and pathogenesis, it has been suggested that the two coronaviral serotypes be considered as distinct viruses [5,6].

The clinical presentation of FIP is a continuum ranging from a predominantly effusive or “wet” form (abdominal or pleural fluid accumulation) to a non-effusive or “dry” form (granulomatous inflammation in organs such as the kidney, brain, eye and lymph nodes) [7]. In both forms of FIP, there may be variable ocular and/or neurologic involvement, although this is less commonly identified with the effusive form [7,8,9]. Once clinical signs of FIP appear, FIP-associated mortality is high without treatment, with one study reporting an average survival time post-presentation of 21 days for cats with effusive FIP and 38 days for cats with non-effusive FIP [10].

The antiviral compounds GC376 and GS-441524 have been extensively explored in both tissue culture and in vivo studies for their ability to inhibit FIPV replication and to treat cats with experimental or naturally acquired FIP [9,11,12,13,14,15,16,17]. Both antiviral agents have variable but generally high success rates in their ability to cure cats with FIP, particularly the effusive form of the disease [9,11,12,13,14,15,16]. GC376 (Anivive Lifesciences), a 3C-like protease (3CL^pro^) inhibitor, targets the viral 3CL protease which is also referred to as the main viral protease (M^pro^). 3CL^pro^ is a cysteine protease and represents an attractive pharmacologic target due to its specific role in viral polyprotein cleavage [17,18,19]. An initial clinical trial that utilized GC376 in 20 cats with naturally occurring FIP documented remission in seven of the 20 cats [11]. A recent retrospective study found that 29 of 30 cats treated with either GC376 or GS-441524 were clinically cured of FIP [20]. GS-441524 is a nucleoside analog with demonstrated efficacy against feline coronavirus [9,13,14] and SARS-CoV-2 [21,22]. In a study of 10 cats that were experimentally infected with FIPV, GS-441524 treatment resulted in the rapid reversal of clinical signs and disease remission in all 10 cats [14]. In a subsequent clinical trial, 31 cats with naturally occurring FIP were treated with GS-441524, which resulted in 25 of 31 cats healthy and disease free at the time of manuscript publication [13]. A large anecdotal study that surveyed owners of cats with FIP being treated with unlicensed GS-441524 found that 96.7% of the cats (n = 380) were alive at the time of publication and 54.0% of those cats considered cured, while another 43.3% were still being monitored as part of the 12-week observation period [9].

The nucleotide analog remdesivir (RDV), a phosphoramidate prodrug of GS-441524, has been demonstrated to be effective in blocking the replication of multiple families of RNA viruses, including *Coronaviridae* (including SARS-CoV, MERS-CoV, FCoV, SARS-CoV-2) [23,24,25,26,27,28], *Paramyxoviridae* (including Nipah virus and Hendra virus) [29], and *Filoviridae* (including Ebola virus and Marburg virus) [30]. RDV is currently utilized for the treatment of hospitalized adults and children over 12 years of age infected with SARS-CoV-2 [31]. However, to the authors’ knowledge, there are no peer-reviewed studies describing the in vivo pharmacokinetics of RDV in cats and no published reports evaluating the antiviral efficacy of RDV against FIPV. However, a non-peer reviewed study recently reported that a compounded form of RDV is being successfully and regularly used in Australia via the intravenous (IV) or subcutaneous (SC) route for the treatment of client-owned cats with naturally occurring FIP [32]. 

Nirmatrelvir is a M^pro^ (or 3CL^pro^) coronaviral protease inhibitor similar to GC376, which has conditional approval for the treatment of mild to moderate COVID-19 in adults and children over 12 years old [33]. For COVID-19 patients, nirmatrelvir is administered orally in combination with ritonavir, and together are marketed under the name Paxlovid (Pfizer) [33]. The protease inhibitor ritonavir is a cytochrome P450 3A4 (CYP3A4) enzyme inhibitor, affecting the absorption and metabolism of other protease inhibitors [34]. The use of low-dose ritonavir in combination with other protease inhibitors results in delayed first-pass metabolism and increased dosing intervals for the primary protease inhibitor [35].

The nucleoside analog molnupiravir (MPV; EIDD-2801) has demonstrated anti-coronaviral activity against both FIPV and SARS-CoV-2 [16,36,37]. Although peer-reviewed trials examining the efficacy of MPV in cats with FIP have not been published, the social media group FIP Warriors anecdotally reports that it is highly effective [38]. To our knowledge, there are no peer-reviewed publications documenting the in vivo pharmacokinetics in cats. The active metabolite of MPV, β-D-N^4^-hydroxycytidine (NHC; EIDD-1931), has also demonstrated broad-spectrum antiviral activity against multiple coronaviruses, including SARS-CoV-2, MERS-CoV, SARS-CoV-1 [39], multiple bat coronaviruses [39], and FIPV in vitro [16]. 

Some systemic manifestations of FIP, including those with neurologic or ocular involvement, appear to be more difficult to treat, possibly as a result of impaired drug penetration into specific anatomic compartments. However, a study documented successful resolution of disease in 3 of 4 cats with neurological FIP using an escalating dose of GS-441524 [40] and a separate anecdotal study based on owner survey responses reported that 161 of 169 cats with neurological or ocular signs could be successfully treated with GS-441524 [9].

Based on the clinical success of combination therapy for the treatment of certain viral diseases including HIV-1 (e.g., combined antiretroviral therapy or CART) [41] and hepatitis C (e.g., Zepatier, Mavyret) [42,43], it is reasonable to explore combined anticoronaviral therapies (CACT) for the treatment of FIP. In a prior in vitro study assessing the efficacy of FIPV antivirals, certain combinations of antiviral drugs demonstrated additive activity against FIPV; however, none of the investigated combinations were more efficacious than GS-441524 or GC376 when used as monotherapy [16]. The study described here builds on the results of this prior study and applies an optimized in vitro bioassay for the assessment of combined anticoronaviral efficacy. Here we compare five antiviral agents used as monotherapies and as CACT in in vitro screening assays utilizing FIPV serotype I (Black I) and serotype II (WSU-79-1146). Additionally, we performed pharmacokinetic (PK) analyses of MPV (oral), GS-441524 (oral), and RDV (oral, intravenous) in specific pathogen free (SPF) cats in vivo to determine a rational feline dosing protocol. 

## 2. Materials and Methods

### 2.1. In Vitro Propagation of FIPV I and II

Serotype I FIPV strain Black I (GenBank EU186072) and culture adapted *Felis catus* whole fetus 4-Cornell University (FCWF-4 CU) [44] cells were generously donated by Dr. Susan Baker (Loyola University Chicago), originally obtained from Cornell University College of Veterinary Medicine (Dr. Edward Dubovi). Black I virus was propagated in FCWF-4 CU cells, cultured in minimal essential media (MEM; Sigma, St. Louis, MO, USA) with 2% fetal bovine serum (FBS; Gemini Bio, West Sacramento, CA, USA) and viral infectivity quantified using a colorimetric bioassay (median tissue culture infectious dose, TCID_50_) as previously described [45]. FIPV serotype II strain WSU-79-1146 (GenBank DQ010921) was propagated in Crandell-Rees feline kidney cells (CRFK; ATCC, Manassas, VA, USA) in complete Dulbecco’s Modified Eagle Medium (DMEM; Gibco, Billings, MT, USA) supplemented with 10% FBS (Gemini Bio) and 1× penicillin/streptomycin (P/S; Gibco). Similar to Black I, viral infectivity was quantified using a colorimetric bioassay (TCID_50_) as previously described [16,45]. Titered virus was aliquoted into 100 or 500 μL volumes and stored at −80 °C until further utilized. 

### 2.2. Determination of Antiviral Efficacy In Vitro

Antiviral compounds were selected based on previous documented efficacy against serotype II FIPV (WSU-79-1146) [14,16,17,46], differing mechanisms of action (nucleoside/nucleotide analog or viral protease inhibitor), and previously documented low to absent evidence of in vitro cytotoxicity in feline cells [16]. GC376 was obtained from Yunjeong Kim (Kansas State University), NHC was sourced from DC Chemicals (dcchemicals.com), and all other antiviral compounds used in the in vitro studies (GS-441524, RDV, nirmatrelvir, and MPV) were sourced from Natural Micron Pharm Tech (NM PharmTech; Tai’an, China). For in vitro experiments, the compounds were received as a powder and stored at 4 °C until reconstituted to 10 mM in DMSO (Sigma Aldrich, St. Louis, MO, USA) and stored at −20 °C.

The half-maximal effective concentration (EC_50_) for each antiviral compound was determined through a colorimetric bioassay to determine the concentration of drug required to obtain 50% of its maximal effect (EC_50_), as a modification of a previously described method [16]. Select FIPV-infected feline cells demonstrate cytopathic effect (CPE), which results in cell injury and loss (or attenuation) of the cell monolayer. Virus-associated CPE can therefore be quantified colorimetrically (loss of absorbance) using a plate reader and can be utilized as a surrogate metric for viral infection. For each assay, a progressive two-fold compound dilution series ranging from 50 to 0.05 μM (10 dilution step series) was performed in tissue culture media [either Minimal Essential Media (MEM)/FBS (FCWF-4 CU cells with Black I) or Dulbecco’s MEM/FBS (CRFK cells with WSU-79-1146)]. FCWF-4 CU (Black I) or CRFK cells (WSU-79-1146) were plated in 96-well tissue culture plates at approximately 25,000 cells per well in their respective media (MEM/FBS or DMEM/FBS, respectively), incubated for 24 h, and then infected with either Black I or WSU-79-1146 virus at a multiplicity of infection (MOI) of 0.1 (one infectious virion for every 10 cells). The virus-infected tissue culture plates were incubated for 1 h and then treated with serially diluted antiviral compounds. Control wells included cells only, and cells with virus but no antiviral agents. All experimental treatments were performed in six well replicates. Culture plates were incubated for 72 additional hours at 37 °C in CO_2_ incubator, fixed with methanol, stained with crystal violet, and scanned for absorbance at 620 nm using an ELISA plate reader (Softmax Pro, Molecular Devices, Silicon Valley, CA, USA). The absorbance data were graphed and the EC_50_ calculated by plotting a non-linear regression equation (inflection point of the dose-response curve) using Prism 9 software (GraphPad, San Diego, CA, USA).

Select antivirals with different mechanisms of action were evaluated for their CACT in blocking the replication of serotype II FIPV (WSU-79-1146). These antiviral combinations included: (i) RDV and GC376, (ii) RDV and nirmatrelvir, and (iii) MPV and nirmatrelvir. Each of these antiviral combinations include a nucleos(t)ide analog (RDV or MPV) along with a viral protease inhibitor (nirmatrelvir or GC376). GS-441524 was not assessed in combination with other compounds as CACT with GS-441524 has been previously reported [16] and this agent faces regulatory challenges with regards to patent protection and advancing to approval for veterinary usage. The combined antiviral efficacy was determined using the colorimetric bioassay and serial 2-fold dilutions of each compound ranging from 50 to 0.05 μM. The EC_50_ for the combined therapy and constituent monotherapies were determined concurrently, as described above. To evaluate CACT for evidence of additive or synergistic effect, the compound fractional inhibition was calculated, and synergy calculations were performed with the freely available software program CompuSyn [47]. To calculate fractional inhibition, absorbance data were converted to “viral activity” using cells with no virus as 0% activity and cells with virus only (no drug) as 100% viral activity. Fractional inhibition for each drug concentration (fraction affected) was then calculated as 1-viral activity and used as input for synergy calculations.

### 2.3. Single Dose Pharmacokinetics of Orally Administered MPV, GS-441524, and RDV and Intravenously Administered RDV

A series of in vivo PK studies to determine the feline-specific pharmacokinetics and metabolism of orally administered MPV, GS-441524, and RDV as well as intravenously administered RDV were performed in healthy, specific pathogen free (SPF) cats sourced from the University of California, Davis’ (UC Davis) Feline Nutrition and Pet Care Center. UC Davis is an AAALAC International accredited institution (#000029), and all experimental procedures were approved by the University’s Institutional Animal Care and Use Committee (UC Davis IACUC #22839 and #22483). Powdered MPV and GS-441524 was sourced from NM Pharmtech, powdered RDV was sourced from MedKoo Biosciences (Morrisville, NC, USA), and the commercially available intravenous formulation of RDV (Veklury^®^, Gilead, Foster City, CA, USA) was utilized for the RDV IV PK study.

Fasted male cats (N = 3 per group) were enrolled per study and were weighed within 24–48 h prior to receiving the study drug to ensure dosing accuracy. Study drugs were administered at the following doses: 10 mg/kg MPV, 25 mg/kg GS-441524, 25 mg/kg RDV (oral), and 7 mg/kg RDV (IV). Orally administered MPV was formulated as excipient-less powder in size 4 gelatin capsules (Spectrum Scientific, Irvine, CA, USA). Orally administered GS-441524 and RDV were formulated as tightly packed powders in gelatin capsules (size 5, XPRS Nutra). Powdered drug was weighed using Fisherbrand weighing paper and a Metler Toledo AX105 DeltaRange scale, and then transferred with a spatula into capsules. 

For intravenously administered RDV, pharmaceutical-grade Veklury^®^ (lyophilized powder, 100 mg per vial) was reconstituted in 19 mL of sterile water and diluted to 5 mg/mL in 0.9% saline prior to slow bolus administration per manufacturer’s instructions over five minutes [48,49]. All cats were male and approximately 1.5 years old.

For all PK studies, a physical examination was performed on each cat at study initiation (timepoint zero). For orally administered MPV, approximately 2.5 mL of whole blood was obtained from a peripheral vein prior to compound administration (time point 0) to establish a baseline complete blood count and serum biochemistry analysis panel for the MPV study. These panels were repeated 48 h post-MPV administration to assess for acute toxicity. After blood collection, the cat was administered an oral gelatin capsule containing the study drug and then mildly sedated with dexmedetomidine (10–40 μg/kg), butorphanol (0.1–0.4 mg/kg), ketamine (1–3 mg/kg), and midazolam (0.1–0.4 mg/kg) administered subcutaneously. Approximately 0.5–1 mL of whole blood was collected at each of the following time points: 0.25, 0.5, 1, 1.5, 2, 3, 6, 9, 12, and 24 h post-administration of MPV. After the 15-min blood collection, an IV catheter was placed into a cephalic vein, secured in place, and flushed with heparinized saline. For the MPV study, one cat (20-024) required a second peripheral blood collection at the 30 min time point due to the IV catheter not being in place or functional at that time point. For all PK studies, after catheter placement, a heparin lock of 0.1 mL of a 10,000 USP per mL solution was placed. At this time, the sedation was reversed with atipamezole intramuscularly (IM) and recovery took approximately 20 min. Prior to all blood collections, approximately 0.5 mL of blood was collected and discarded to remove the heparin lock. After each blood sample was collected, a fresh 0.1 mL heparin lock was placed into the catheter. Blood collection at the 24 h time point was collected via manual restraint and from the jugular vein. 

For orally administered MPV, at each collection time point the blood sample was incubated for 5 min at room temperature to allow for clotting and then centrifuged at 12,000× *g* for 10 min to separate the serum fraction from the blood cells. One hundred microliters of serum were aliquoted into a 1.5 mL microcentrifuge tube containing 300 μL of acetonitrile to halt compound metabolism by serum esterases and immediately placed in an insulated container with dry ice for temporary storage and freezing. Samples were subsequently stored at −80 °C prior to liquid chromatography tandem-mass spectrometry (LCMSMS) analysis. 

GS-441524 is the main systemically circulating metabolite of RDV [48,50] and was thus quantified in plasma for both GS-441524 and RDV studies. For orally administered GS-441524 and RDV, approximately 0.5–1 mL of whole blood was collected at the following timepoints: 0.5, 1, 3, 6, 8, and 24 h post-dose. The encapsulated study drug was orally administered at timepoint zero and then each cat immediately sedated with a combination of ketamine (1–3 mg/kg), butorphanol (0.1–0.4 mg/kg), and midazolam (0.1–0.4 mg/kg) followed by catheter placement. For intravenously administered RDV, approximately 0.5–1 mL of whole blood was collected at the following timepoints: 0.0833, 0.5, 1, 3, 6, 8, and 24 h post-dose. The cats were immediately sedated, followed by catheter placement, and then IV RDV was administered. Whole blood sampled at each time point was centrifuged at 500× *g* for 5 min, the plasma transferred to a clean 1.5 MCT tube, and centrifuged a second time at 1000× *g* for 5 min. The resulting plasma was stored at −20 °C prior to LCMSMS analysis.

### 2.4. LCMSMS Quantitation of Serum MPV Concentrations and Pharmacokinetic Analysis

Analysis of serum MPV and its main metabolite, NHC (EIDD-1931), was performed by modification of a previously validated and published method for human samples [51]. Briefly, stock solution of MPV (EIDD-2801; NM Pharmtech, 99.0% purity) and NHC (EIDD-1931; MedChemExpress, Monmouth Junction, NJ, USA, 99.73% purity) were prepared at 2 mg/mL in methanol and stored at −80 °C. Stock solutions were further diluted in methanol to produce calibration standards for a low concentration curve at 1, 5, 10, 50, and 100 ng/mL of MPV and NHC combined and a high concentration curve at 250, 500, 750, 1000, 2500 and 5000 ng/mL of NHC alone. Calibration curves were generated by fortification of blank feline serum obtained from the clinical pathology laboratory at the UC Davis Veterinary Teaching Hospital (pooled from 6 individuals). Quality control samples were generated at 10 and 100 ng/mL (MPV and NHC combined) and at 500 ng and 2500 ng (NHC only). Calibrator and QC serum samples (100 µL) were added to 300 µL acetonitrile (as was done for samples in the in vivo PK study) and then all calibrators, QCs and unknown samples were vortex mixed for 5 min followed by centrifugation at 10,000× *g* for 5 min at 4 °C. After centrifugation, 275 µL of supernatant was removed, transferred to clean 2 mL microcentrifuge tubes, and evaporated to dryness (approximately 1 h) in a SpeedVac (Eppendorf, Hamburg, Germany). Samples were reconstituted in 100 µL of starting HPLC mobile phase (98% 1 mM ammonium acetate pH 4.3 and 2% acetonitrile containing 1 mM ammonium acetate). For analysis, 5 µL was injected into the LC-MS/MS system. 

### 2.5. Mass Spectrometry and Liquid Chromatography Conditions

Negative ion electrospray ionization mass spectra were obtained on a Sciex 6500+ Q-TRAP triple quadrupole mass spectrometer (AB Sciex LLC, Framingham, MA, USA) with a turbo ionspray source coupled to the Sciex ExcionLC™ UHPLC system with a cooled (15 °C) autosampler. Chromatographic separation was carried out on a Luna^®^ Phenyl-hexyl 3.0 µm column (50 × 2.0 mm) with a filter frit guard column (both from Phenomenex, Inc., Torrance, CA, USA). A gradient mobile phase was employed consisting of 1 mM ammonium acetate pH 4.3 (mobile phase A) and acetonitrile containing 1 mM ammonium acetate (mobile phase B). Separation was carried out by holding mobile phase B constant at 2% for 1.2 min, increasing linearly to 90% at 2.2 min, holding at 90% until 3.5 min, decreasing linearly back to 2% from at 3.75 min and equilibrating at 2% until 5.0 min. Analytes were identified by monitoring the ion transitions for MPV (*m/z* 328.1 → 126.0 and 168.1) and for NHC (*m/z* 257.9 → 125.9 and 107.9). Quantitation was performed by linear regression of analyte peak areas in unknown samples with calibrator samples using 1/x^2^ weighting. 

### 2.6. LC-MS/MS Quantification of GS-441524 in Plasma

Plasma levels of GS-441524 were analyzed at Covance, Inc. (Princeton, NJ, USA) on a fee-for-service basis using a liquid chromatography mass spectrometry (LC-MS/MS) assay described previously [48]. Briefly, For plasma samples, a 25 μL aliquot was treated with 200 μL of 100% acetonitrile with 20 nM 5-(2-aminopropyl)indole (5-IT; as the internal standard). Samples were filtered through an Agilent Captiva 96-well 0.2 μm filter plate. Filtered samples were then dried down completely for approximately 30 min and reconstituted with 250 μL water. A 10 μL aliquot was then injected for LC-MS/MS analysis. Analytes were separated on a Phenomenex Synergi Hydro-RP 30A column (150 Å~2.0 mM, 4.0 μm) at 25 ÅãC using a Waters Acquity Ultra Performance LC (Waters Corporation, Milford, MA, USA), a flow rate of 0.26 mL/min, and a gradient from mobile phase A (Water containing 0.2% formic acid) and 1% mobile phase B (acetonitrile/water, 95:5, containing 0.2% formic acid) over 4.5 min. MS/MS analyses used a Waters Xevo TQ-S triple quadrupole mass spectrometer (Waters Corporation) with a electrospray probe and analytes were detected in positive ion mode with a multiple reaction monitoring (MRM) method (*m*/*z* 292→163 for GS-441524 and 393 → 261 for 5-IT). Plasma concentrations were determined using an 8-point calibration curve spanning a concentration range of over three orders of magnitude (6.9 to 3430 nM). Quality control samples were run at the beginning and end of the run to ensure accuracy and precision within 20%.

### 2.7. Pharmacokinetic Analysis

Pharmacokinetic parameters for both MPV and NHC were examined following oral dosing of MPV and were estimated by noncompartmental analysis using the commercially available software program Phoenix WinNonlin v8.3 (Certara Inc., Princeton, NJ, USA) and estimation of T_1/2_ was made using three to four timepoints. For GS-441524 and RDV studies, data from all were analyzed using PKSolver 2.0 using non-compartmental parameters using two elimination timepoints and graphs were generated using GraphPad Prism 8.

## 3. Results

### 3.1. All of the Antiviral Agents Effectively Block the In Vitro Replication of Both Serotype I and II FIPV as Monotherapies

**Figure 1:**
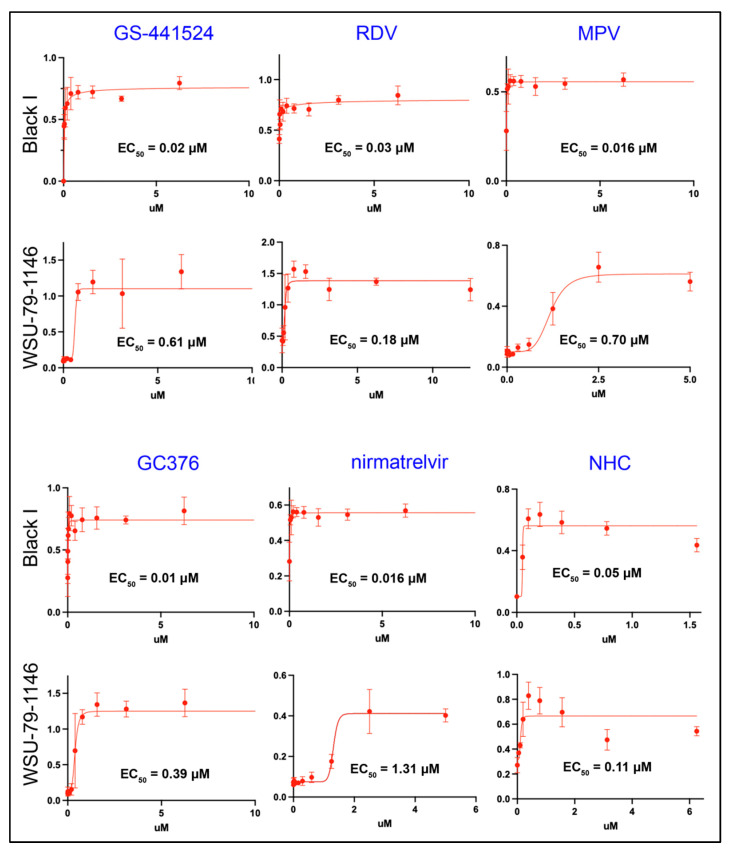
Compound inhibition of serotype I and II FIPV as monotherapies. Half-maximal effective concentration (EC_50_) values for the tested nucleoside analogs and protease inhibitors against either Black I (serotype I FIPV) or WSU-79-1146 (serotype II FIPV) and corresponding non-linear regression analyses; all plate absorbance values obtained at 620 nm. For each compound concentration, the absorbance value is the mean of 6 independent wells and error bars reflect the standard deviation. Serial dilutions of each compound were performed to determine the EC_50_.

A plate-based colorimetric bioassay was used to determine the antiviral EC_50_ values for the nucleoside analogs GS-441524, RDV, MPV, NHC, and the protease inhibitors GC376 and nirmatrelvir against both FIPV I and II. Used as monotherapies, all of the tested compounds were determined to be potent inhibitors of FIPV I and II replication with EC_50_ values close to 1 μM or less (Figure 1). GC376 was determined to be the compound with the lowest EC_50_ (highest antiviral efficacy) for FIPV I (0.01 μM) while the NHC had the lowest EC_50_ for FIPV II FIPV (0.11 μM). All of the tested antiviral compounds consistently demonstrated lower EC_50_ values for FIPV I relative to FIPV II with nirmatrelvir demonstrating the greatest differential efficacy (nearly 100-fold). Evidence of CPE was not identified in the control wells (cells only) and marked CPE (clearance of the well monolayer) was identified within wells with cells and virus only (no antiviral agent).

### 3.2. Select Antiviral Agents Have a Synergistic Effect When Used in Combination

**Figure 2:**
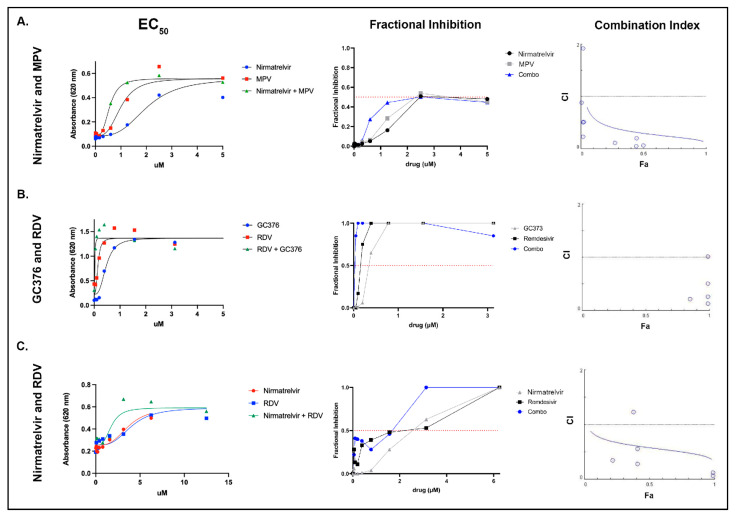
EC50, fractional inhibition values, and isobologram plots (combination index) representing the three evaluated compound combinations. Left column visually demonstrating individual nonlinear regression curves overlain with combination nonlinear regression curves for the three listed combinations. EC_50_ values: (**A**) Nirmatrelvir alone—1.313 μM, MPV alone—0.693 μM, nirmatrelvir combined with MPV—0.5640 μM. (**B**) GC376 alone—0.406 μM, remdesivir alone—0.181 μM, GC376 combined with remdesivir—0.046 μM. (**C**) remdesivir alone—6.416 μM, nirmatrelvir alone—2.936 μM, and RDV combined with nirmatrelvir—1.670 μM. The middle column shows fractional inhibition curves demonstrating the increased fractional inhibition for antiviral combinations at the corresponding drug concentrations. The right column includes isobologram plots that illustrate CI values generally below 1 for all three antiviral compounds, consistent with compound synergy (horizontal line). Fraction affected (Fa) shown on x axis.

Individual antiviral combinations were selected based on differing mechanisms of action (e.g., nucleoside analog paired with a protease inhibitor). NHC (EIDD-1931) was not utilized in combination antiviral assessment based on a progressive reduction in absorbance (presumed cytotoxicity) appreciated above 1.5 μM in the monotherapy assessment above (Figure 1, NHC). EC_50_ values were as follows: (A) nirmatrelvir alone—1.313 μM, MPV alone—0.693 μM, nirmatrelvir combined with MPV—0.5640 μM. (B) EC_50_ values: GC376 alone—0.406 μM, remdesivir alone—0.181 μM, GC376 combined with remdesivir—0.046 μM. (C) EC_50_ values: remdesivir alone—6.416 μM, nirmatrelvir alone—2.936 μM, and RDV combined with nirmatrelvir—1.670 μM (Figure 2, left column). 

Similarly, fractional inhibition curves are shifted left for every drug combination evaluated except for nirmatrelvir combined with RDV between 1 and 2 μM (Figure 2, middle column). Further, these curves show that the combination of GC376 and remdesivir reached almost complete fractional inhibition at the lower range of compound concentration, nirmatrelvir combined with MPV only reached approximately 50% fractional inhibition, and nirmatrelvir combined with RDV approached 100% fractional inhibition at approximately 2.5 μM.

Compound synergy was calculated and determined to be present for the following antiviral combinations: (1) nirmatrelvir with MPV, (2) GC376 with RDV, and (3) nirmatrelvir with RDV. Synergy was indicated by combination index (CI) values of <1 for the three antiviral combinations (Figure 2, right column).

### 3.3. Pharmacokinetics of Oral MPV in Cats

An abbreviated method validation was performed to ensure the method adapted from human plasma was reproducible in feline serum. Blank feline serum did not have a signal at the retention time for the analytes. The calibration curve (n = 11 non-zero concentrations) was linear with an r > 0.99 and accuracy of each concentration within 10% of nominal. The quality control (QC) samples had accuracies within 15% of nominal and precision of calibrators and QC samples was within 10%. A single freeze–thaw cycle was performed at 50 ng/mL (NHC and MPV) and 1000 ng/mL (NHC) and demonstrated stability of NHC and MPV (when combined with acetonitrile) in feline serum with calculated concentrations within 5% or original concentration. 

We found no evidence of MPV-associated acute organ toxicity in any of the cats based on the pre- and post-treatment complete blood count (CBC) and serum biochemistry panels (Supplementary Tables S1–S3). However, all three of the MPV-treated cats demonstrated variable signs of nausea, including hypersalivation and/or vomiting, after oral administration of MPV (10 mg/kg). Cat 20-024 demonstrated hypersalivation at 1.5 h post administration, had a small amount of vomitus at 2 h, and maintained hypersalivation until 3 h post-administration, at which point the cat regained an appetite. Cat 20-025 exhibited lip licking between 1.5- and 2-h post-administration and lacked an appetite until approximately 6 h post-administration. Cat 20-031 had marked hypersalivation at 1-h post-administration and regained an appetite at 5.5 h post-administration of MPV. 

**Figure 3:**
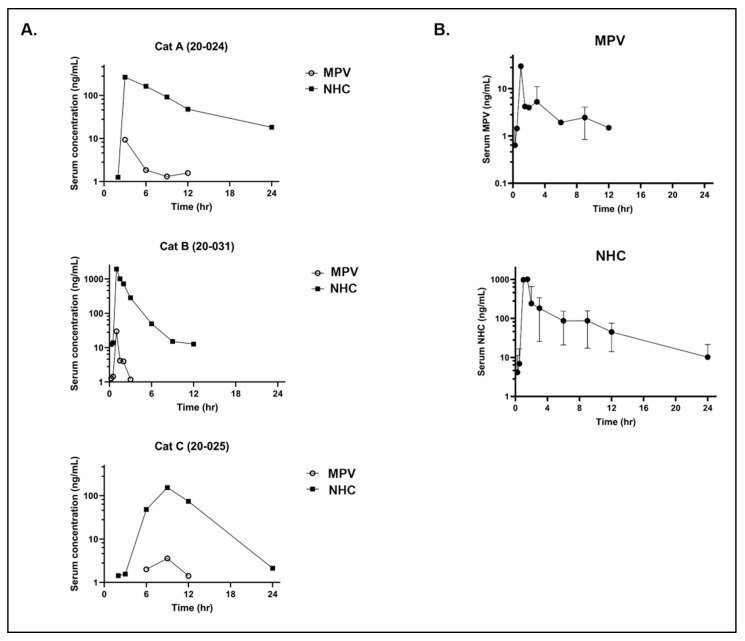
Serum detection of prodrug MPV and the metabolite NHC over a 24-h time period in cats. (**A**) Detection of MPV and NHC in the serum (ng/mL) of each individual cat over 24 h. (**B**) Combined detection of MPV and NHC in three cats over 24 h.

The MPV prodrug was detected in the serum of all three cats at low levels in the first 12 h post-administration while the NHC metabolite was detected at much higher concentrations between 12 to 24 h post-administration. Marked variability in detected NHC levels was present between individual cats (Figure 3A). For cat 20-024, the peak detection level for NHC and MPV occurred at the 3-h time point with NHC detected at a 10-fold greater level than MPV. For cat 20-031, peak detection levels occur at the 1.5-h time point, with MPV nearly 100-fold below NHC. The peak detection levels occurred for cat 20-025 at the 9-h time point with NHC detected nearly 100-fold over MPV. NHC was more consistently and quantifiably detected over the 24-h time period, aside for cat 20-031, for which NHC was no longer detected (based on limit of detection) after 12 h post-administration. MPV, however, was not consistently detected at the early time points nor after the 12-h time point. 

In aggregate, the data for the three cats (Figure 3B) indicate that peak detection time points for MPV and NHC occur at approximately 1.5 and 2 h post MPV administration. MPV was not detected after the 12-h time point, consistent with rapid metabolic conversion to NHC in vivo.

For orally administered MPV at 10 mg/kg, the PK parameters for the parent drug (MPV) showed a mean maximal concentration of drug in plasma (C_max_) of 14.237 ng/mL (0.043 μM; SD ± 13.781) and an average exposure [measured as area under the curve (AUC_0-t_) where t = last measurable concentration] value of 27.546 h*ng/mL (0.085 μM*h) (Table 1). 

For orally administered MPV at 10 mg/kg, the PK parameters for the NHC metabolite demonstrated an average plasma C_max_ of 790 ng/mL (3.05 µM; SD ± 1006) at a corresponding average time of maximum concentration (T_max_) of 4.3 h with an average AUC value of 1779.7 h*ng/mL (6.87 μM*h) (Table 2). In all three cats, serum exposure reached levels greater than the NHC EC_50_ (0.05 μM for Black I, which is equivalent to 12.9 ng/mL) early in the 24-h time period, and in one cat fell to approximately the level of the EC_50_ by the 12-h time point. 

### 3.4. Pharmacokinetics of GS-441524 after Administration of GS-441524 or RDV in Cats

No clinical abnormalities were noted in the GS-441524 or IV RDV group. However, mild ptyalism, nausea, and lethargy were observed in 2 of 3 cats after oral RDV treatment; these symptoms eventually subsided, and the cats were determined to be clinically normal by the end of the day (~12 h later). 

**Figure 4:**
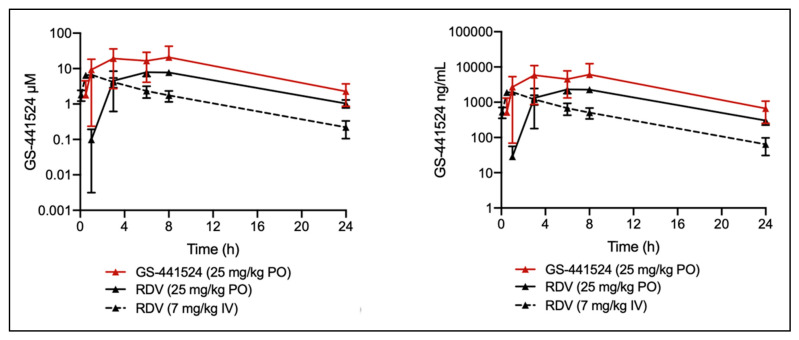
Detection of the metabolite, GS-441524, in the plasma of cats administered either oral GS-441524 (25 mg/kg, red line), oral RDV (25 mg/kg, solid black line), or intravenous RDV (7 mg/kg, dashed black line) over 24 h. Data are shown as the mean ± SD (N = 3) per drug cohort.

Across all treatment groups, plasma levels of GS-441524 were high (Figure 4). In the oral GS-441524 cohort, plasma concentrations reached an average C_max_ value of 10,290 ng/mL (35 µM) (Table 3). The time-of-maximum concentration (T_max_) occurred at 3 h for two of the cats; however, one cat exhibited a T_max_ at 8 h, which could be attributed to fractious behavior during dose administration. Drug exposure was AUC_0-24_ of 89,708 h*ng/mL (308 µM*h) and a 24 h concentration (herein referred to as C_24_) of approximately 664 ± ng/mL (2.3 ± 1.4 µM).

In the oral RDV cohort, GS-441524 exhibited an average plasma C_max_ of 2480 ng/mL (7.9 µM) at a corresponding T_max_ of 6 h. The average AUC_0-24_ value was found to be 32,038 h*ng/mL (110 µM*h; average C_24_ = 302 ± 75.7 ng/mL, 1 ± 0.26 µM) (Table 4). Finally, in the IV RDV cohort, GS-441524 exhibited an average plasma C_max_ of 2003 ng/mL (6.9 µM) at a corresponding T_max_ of 0.83 h and an average AUC_0-24_ value of 13,162 h*ng/mL (45 µM*h; average C_24_ = 64 ± 32 ng/mL, 0.22 ± 0.11 µM) (Table 5). In general, plasma exposure to GS-441524 was above the EC_50_ for all treatment groups, with 0.22–2 µM of GS-441524 remaining 24 h post-treatment. Average percent oral bioavailability (F%) of GS-441524 when either GS-441524 or RDV were orally administered was high, exceeding that observed for IV administered GS-441524 (Table 3 and Table 4) [14].

## 4. Discussion

Through a series of in vitro assays, these data demonstrate the feasibility of CACT for the treatment of FIP in cats based on evidence of synergy in select antiviral combinations in vitro. We have also provided data on the in vivo pharmacokinetics and metabolism in cats of MPV administered orally, GS-441524 administered orally, and RDV administered orally or intravenously. Further, we have documented the in vivo detection of the MPV active metabolite, NHC, over a 24-h time period. 

A selection of antiviral compounds (nucleoside analogs and viral protease inhibitors) with either previously determined evidence of antiviral efficacy against FIPV serotype II or promising anecdotal evidence of antiviral efficacy were comparatively evaluated using an in vitro colorimetric plate bioassay in order to determine efficacy against FIPV serotype I (Black I) and serotype II (WSU-79-1146) [11,13,14,16]. We found that all of the evaluated compounds, with the exception of nirmatrelvir, had EC_50_ values below 1 μM when tested against FIPV serotype II (WSU-79-1146). For serotype I FIPV, all compounds consistently demonstrated greater efficacy (lower EC_50_ values) relative to serotype II FIPV (2- to 84-fold). The cause of this consistent difference in EC_50_ values between the two viral serotypes was not definitively determined, however, it may reflect differences in viral growth kinetics and a differential temporal manifestation of CPE between the two viruses and their respective permissive cell lines. The EC_50_ values for GC376, GS-441524, MPV, and NHC against WSU-79-1146 propagated in CRFK cells were comparable to values previously determined in our laboratory (0.39, 0.61, 0.7 and 0.11 μM, respectively) [16]. A prior study reported an EC_50_ for GC376 against FIPV II of 0.9 μM, relatively comparable to the findings reported here (0.39 μM) [52]. The EC_50_ for GS-441524 against serotype II FIPV has also been previously reported to be 0.78 μM [14], similar to the results described here (0.61 μM). 

The antiviral compounds were evaluated in vitro at concentrations ranging up to 50 μM. For NHC, there was a notable and progressive decrease in well absorbance values at concentrations greater than 1.5 μM in FCWF-4 CU cells, consistent with direct compound-associated cell injury (cytotoxicity). NHC-associated cytotoxicity has been demonstrated in other cells lines with a 50% cytotoxic concentration (CC_50_) of 7.5 μM when used in the CEM/C1 human cell line [53]. NHC-associated cytotoxicity has also been documented in CRFK cells using a fluorescence methodology [16]. More in-depth evaluations of potential NHC cytotoxicity in both in vitro and in vivo experiments are warranted, including evaluation of repeat dosing and long-term monitoring of systemic parameters in cats, such as CBC and serum biochemistry panels. 

Based on the monotherapy results demonstrating a predictable and consistently lower EC_50_ for antivirals against the Black I tissue culture strain relative to WSU-79-1146, we performed select combination experiments in vitro against WSU-79-1146 (CACT). The three evaluated combinations were (1) nirmatrelvir combined with MPV, (2) GC376 combined with RDV, and (3) RDV combined with nirmatrelvir. All three antiviral combinations demonstrated a nonlinear regression curve shift to the left indicating increased efficacy when the antivirals were used in combination. Consistent with the improved EC_50_ values for antiviral combinations, there was a similar left shift in fractional inhibition (the fraction of cells protected from death appreciated via absorbance). Interestingly, the combination of nirmaltrelvir and MPV only reached approximately 50% fractional inhibition, suggesting that this combination may be less attractive with regards to overall antiviral efficacy. GC376 combined with RDV was the most effective combination when considering fractional inhibition, as this combination approached 100% fractional inhibition at the lowest range of compound concentrations utilized. Combination index (CI) values were also plotted in order to identify evidence of compound synergy at different fractions affected (indicating the fraction of cells inhibited after drug exposure). The CI values were <1 at almost all data points, consistent with compound synergy. Antiviral synergy suggests that CACT may offer an alternative approach to the use of monotherapies for the treatment of FIP. Further investigations are needed to define if CACT may result in increased cure rates for FIP, particularly for those cats with neurologic or ocular involvement or those cats experiencing therapeutic relapse. 

The GS-441524 dose was selected based on earlier findings that once-daily oral administration at 25 mg/kg yielded curative efficacy in cats with FIP (personal communication, unpublished), although dosages in the literature and in practice vary [9]. Oral RDV dosage was selected based on a combination of peer-reviewed publications documenting differences in pharmacokinetics and oral bioavailability between RDV and GS-441524 in mice as well as non-peer reviewed recommendations put forth by veterinarians in Australia who have been successfully treating cats with FIP [32,54]. Although the molecular weight (MW) of RDV is approximately twice that of GS-441524, bioavailability differences between the two compounds negate these differences in MW and require relatively higher doses of RDV [54]. This is supported by comparing pharmacokinetics data of GS-441524 and RDV in vitro and after oral administration in mice [54]. The IV RDV dose corresponds to the allometrically scaled 200 mg first dose used for treating COVID-19 human patients [55].

Although we found no evidence of acute toxicity in the MPV-treated cats based on complete blood count and blood chemistry assessments, all three cats had clinical signs consistent with nausea for several hours post administration. This suggests an association between administration of MPV and nausea in cats. However, all cats were sedated after administration of the drug in order to place an indwelling IV catheter for subsequent blood collections so nausea secondary to sedation cannot be completely ruled out. The cats were also fasted prior to receiving MPV, which might play a role in the development of nausea. In a first-in-human study with MPV, a food effect on the rate of absorption was observed; however, the therapeutic exposures under fasted and fed states were comparable [56]. Due to the emergence of these treatment-related events, a higher oral dose of RDV equimolar to 25 mg/kg GS-441524 was not investigated.

Similar to what has been identified in humans, we found that MPV is rapidly metabolized in feline plasma to the active metabolite, NHC [56]. We detected the NHC metabolite at early timepoints and at markedly higher levels than the administered prodrug, MPV. In all three cats, we established that serum exposure rapidly reached levels greater than the NHC EC_50_, and in one cat fell to approximately the level of the EC_50_ by the 12-h time point. We also found marked interindividual variability in MPV metabolism. A definitive cause for the metabolic variability between individual cats is presently undetermined but possible causes could include minor variability in the timing between centrifugation of individual whole blood samples between cats and the timing of the transfer of feline serum into acetonitrile. Variation in genetic polymorphisms related to metabolic pathways, such as the cytochrome P450 system, might also play a role in this variability [57,58]. One study focused on the administration of clopidogrel in cats found that high interindividual variability was associated with sex and cytochrome P450 2C genetic polymorphisms [59]. With a determined NHC half-life of approximately 3 h, we predict that twice daily dosing should not lead to accumulation of drug within the plasma. 

The PK data demonstrate that GS-441524 and RDV have sufficient oral bioavailability and that this route of administration can yield therapeutic plasma levels of GS-441524 for treatment of FIP. Peak plasmatic concentrations of GS-441524 were > 30 µM after oral administration of GS-441524 at 25 mg/kg with no treatment-related adverse events; this is approximately 3-fold higher than the C_max_ when GS-441524 is subcutaneously administered at the 5 mg/kg (~10 µM). Multiple clinical studies have used ~5 mg/kg as the therapeutic dose, although higher doses are now commonly used with an approximate range of 2–16 mg/kg [9,13,14]. A significant clinical advantage for oral administration of GS-441524 is the elimination of injection site reactions associated with the pH 1.5 solution required for solvating the compound. This suggests that oral GS-441524 may be more amenable for long-term treatment of neurological or ocular FIP [13,40]. The data indicate that oral administration of RDV for systemic delivery of GS-441524 is feasible. When cats were administered 25 mg/kg RDV orally, plasma C_max_ values of GS-441524 were approximately 10 µM, which is similar to that observed with 5 mg/kg subcutaneously administered GS-441524. Further investigation into the nausea and ptyalism observed in 2 of 3 cats in the oral RDV group is warranted and loading/maintenance dose modification similar to that used for RDV in humans should be explored to circumvent these treatment-related events [55].

While some of the studied antivirals have prior demonstrated success in the treatment of FIP in vivo [9,11,13,14] or hold promise based on in vitro data [14,16], the differing forms of FIP and clinical presentations indicate that a “one-size-fits-all” monotherapy approach to treatment may be overly simplistic. Determination of optimal combination therapies and consideration of a compound’s ability to penetrate into certain viral anatomic niches (e.g., CNS and/or ocular compartments) should be determined in order to optimize therapeutic approaches to cats that experience therapeutic relapse. Although multiple non-peer reviewed clinical strategies for treating FIP are readily available, there are limited rigorously designed clinical trials focused on the treatment of cats with naturally occurring FIP in the peer-reviewed literature. Further, there are little to no PK data for many of these antiviral agents and essentially no information regarding the penetration of various compounds into feline tissues. Such pharmacologic information serves as a critical and rational foundation for designing clinical trials aimed at treating cats with refractory FIP.

Additional in vivo pharmacokinetic studies (CACT assessment), assessment of compound penetration into tissues and rigorously designed and peer reviewed clinical trials focused on both monotherapies and CACT are needed to further optimize and individualize the successful treatment of this historically fatal disease in cats, now demonstrated to be amenable to a variety of antiviral therapies.

## Figures and Tables

**Table 1:** Pharmacokinetic parameters for parent drug (MPV) from noncompartmental analysis following a 10 mg/kg oral dose of molnupiravir in three cats.

Cat ID	C_max_ (ng/mL; µM)	AUC (h*ng/mL)	Vz/F (L/kg)	Cl/F (L/h/kg)	MRT (h)
20-024	9.3 (0.03)	39.6	1101.7	209.0	4.4
20-025	3.6 (0.01)	21.7	1229.4	381.8	8.1
20-031	29.8 (0.09)	21.4	312.4	449.6	1.2
Mean	14.2 (0.04)	27.5	881.1	346.8	4.6
SD	13.8	10.4	405.5	101.3	3.4

C_max:_ maximum serum concentration; AUC: area under the MPV serum concentration-time curve; Vz (apparent volume of distribution) and Cl (apparent clearance) are reported as Vz/F and Cl/F as only oral dose data were collected. MRT: mean residence time.

**Table 2:** Pharmacokinetic parameters for metabolite (NHC) from noncompartmental analysis following a single 10 mg/kg dose of molnupiravir in three cats.

Cat ID	C_max_ (ng/mL; μM)	T_max_ (h)	K_el_ (1/h)	T_1/2_ (h)	AUC (h*ng/mL)	MRT (h)
20-024	265 (1.02)	3.0	0.12	6	1709.47	8.33
20-025	154 (0.15)	9.0	0.29	2.4	950.03	10.51
20-031	1950 (7.52)	1.0	0.26	2.65	2679.57	2.46
Mean	790 (3.05)	4.3	0.2	3.1	1779.7	7.1
SD	1006	4.2	0.1	1.2	866.9	4.2

K_el_: terminal elimination rate; T_1/2_: half-life. Half-life is reported as the harmonic mean and pseudo standard deviation.

**Table 3:** Pharmacokinetic parameters for GS-441524 after a single oral dose of GS-441524 (25 mg/kg) in 3 cats.

Cat ID	C_max_ (ng/mL; μM)	T_max_ (h)	T_1/2_ (h)	AUC (h*ng/mL)	F% *
20-036	13,300 (45.66)	8	4.5	132,232	235
20-044	9100 (31.24)	3	8.8	54,756	100
20-046	8470 (29.08)	3	5.6	82,135	145
Mean	10,290 (35.33)	4.7	6.3	89,708	160
SD	2625 (9.01)	2.9	2.2	39,028	69

* Relative to 5 mg/kg IV GS-441524 [14].

**Table 4:** Pharmacokinetic parameters for GS-441524 after a single oral dose of RDV (25 mg/kg) in 3 cats.

**Cat ID**	**C_max_** **(ng/mL;** **μM)**	**T_max_** **(h)**	**T_1/2_** **(h)**	**AUC** (h*ng/mL)	F% *
20-003	2100 (7.21)	8	5.9	31,747	120
20-024	2390 (8.21)	8	4.6	28,252	103
20-031	2950 (10.13)	6	6.0	36,116	137
Mean	2480 (8.51)	7.3	5.5	32,038	120
SD	432 (1.48)	1.2	0.8	4077	17

* Relative to 5 mg/kg IV GS-441524 [14].

**Table 5:** Pharmacokinetic parameters for GS-441524 after a single IV dose of RDV (7 mg/kg) in 3 cats.

Cat ID	C_max_ (ng/mL; μM)	T_max_ (h)	T_1/2_ (h)	AUC (h*ng/mL)
20-045	1730 (5.94)	0.5	5.3	14,271
21-001	1960 (6.73)	1	5.7	15,291
21-004	2320 (7.97)	1	4.5	9924
Mean	2003 (6.88)	0.83	5.2	13,162
SD	297 (1.0)	0.29	0.6	2850

## Data Availability

Not applicable.

## Supplementary Materials

The following supporting information can be downloaded at: https://www.mdpi.com/1999-4915/14/11/2429/s1, Table S1: Baseline and 48 h post-MPV complete blood count and biochemistry panel for 20-024; Table S2: Baseline and 48 h post-MPV complete blood count and biochemistry panel for 20-025; Table S3: Baseline and 48 h post-MPV complete blood count and biochemistry panel for 20-031.
