# Correction: Cook et al. An Optimized Bioassay for Screening Combined Anticoronaviral Compounds for Efficacy against Feline Infectious Peritonitis Virus with Pharmacokinetic Analyses of GS-441524, Remdesivir, and Molnupiravir in Cats. *Viruses* 2022, *14*, 2429

**DOI:** 10.3390/v16030397

**Published:** 2024-03-04

**Authors:** Sarah Cook, Luke Wittenburg, Victoria C. Yan, Jacob H. Theil, Diego Castillo, Krystle L. Reagan, Sonyia Williams, Cong-Dat Pham, Chun Li, Florian L. Muller, Brian G. Murphy

**Affiliations:** 1 Department of Pathology, Microbiology, and Immunology, School of Veterinary Medicine, University of California-Davis, Davis, CA 95616, USA; ldcastillo@ucdavis.edu (D.C.); sywilliams@ucdavis.edu (S.W.); bmurphy@ucdavis.edu (B.G.M.); 2 Department of Surgical and Radiological Sciences, School of Veterinary Medicine, University of California-Davis, Davis, CA 95616, USA; lwittenburg@ucdavis.edu; 3 Department of Cancer Systems Imaging, University of Texas MD Anderson Cancer Center, Houston, TX 77054, USA; yan22v@mtholyoke.edu (V.C.Y.); cpham3@mdanderson.org (C.-D.P.); cli@mdanderson.org (C.L.); 4 Office of Research, Campus Veterinary Services, University of California-Davis, Davis, CA 95616, USA; jhtheil@ucdavis.edu; 5 Department of Veterinary Medicine and Epidemiology, School of Veterinary Medicine, University of California-Davis, Davis, CA 95616, USA; kreagan@ucdavis.edu; 6 Sporos Bioventures, @JLABS Suite 201, 2450 Holcombe Blvd, Houston, TX 77021, USA; aettius@aol.com

## Error in Table

In the original publication [1], incorrect numerical data were entered into the last three numbers in the AUC column of Table 5 and this mistake was not recognized by our review team. This error was identified by Sally Coggins who brought it to our attention. Corrected Table 5 appears below.

**Table 5:** Pharmacokinetic parameters for GS-441524 after a single IV dose of RDV (7 mg/kg) in 3 cats.

Cat ID	C_max_ (ng/mL; μM)	T_max_ (h)	T_1/2_ (h)	AUC (h*ng/mL)
20-045	1730 (5.94)	0.5	5.3	14,271
21-001	1960 (6.73)	1	5.7	15,291
21-004	2320 (7.97)	1	4.5	9924
Mean	2003 (6.88)	0.83	5.2	13,162
SD	297 (1.0)	0.29	0.6	2850

## Text Correction

With regard to the correction of Table 5, a correction has been made to Section 3.4, Paragraph 3.

“Finally, in the IV RDV cohort, GS-441524 exhibited an average plasma Cmax of 2003 ng/mL (6.9 μM) at a corresponding Tmax of 0.83 h and an average AUC_0-24_ value of 13,162 h*ng/mL (45 μM*h; average C24 = 64 ± 32 ng/mL, 0.22 ± 0.11 μM) (Table 5).”

The authors state that the scientific conclusions are unaffected. This correction was approved by the Academic Editor. The original publication has also been updated.
