# Supplementary material for: An Optimized Bioassay for Screening Combined Anticoronaviral Compounds for Efficacy against Feline Infectious Peritonitis Virus with Pharmacokinetic Analyses of GS-441524, Remdesivir, and Molnupiravir in Cats
Source: Viruses. 2022 Nov 1;14(11):2429. doi: 10.3390/v14112429 (PMC9697187; doi:10.3390/v14112429)
Supplement: Supplementary file 1 [file viruses-14-02429-s001.zip › viruses-1958686-supplementary.pdf]

Table S1. Baseline and 48 hour post-MPV complete blood count and biochemistry panel for 20-024.

ID #		WBC (K/ul)	Absolute Neutrophil cells (K/ul)	Absolute Lymphocyte cells (K/ul)	Absolute Monocyte cells (K/ul)	Absolute Eosinophil cells (K/ul)	Absolute Basophil cells (K/ul)	Neutrophil %	Lymphocyte %	Monocyte %	Eosinophil %	Basophil %	RBC (M/ul)	Hemoglobin (g/dL)	Hematocrit %	MCV (fL)	MCH (pg)	MCHC (g/dL)	RDW %	Platelets (K/uL)	MPV (fL)	Presence of clots
20-024	*Baseline*	7.14	3.83	3.08	0.13	0.10	0.01	53.66	43.11	1.76	1.36	0.11	11.46	14.4	46.9	40.9	12.6	30.7	19.8	175	11.1	none
	48 hour	8.66	5.79	2.50	0.09	0.26	0.01	66.88	28.91	1.07	3.00	0.13	9.54	11.7	39.8	41.7	12.3	29.4	19.9	241	11.8	none

ID #		Alanine Transaminase U/L	Aspartate Transaminase U/L	Albumin g/dL	Alkaline Phosphatase U/L	Amylase U/L	Blood Urea Nitrogen mg/dL	Calcium mg/dL	Chloride mmol/L	Creatinine mg/dL	Glucose mg/dL	Potassium mmol/L	Sodium mmol/L	Phosphorus mg/dL	Total Bilirubin mg/dL	Total Protein g/dL	Hemolysis:
20-024	*Baseline*	62.4	79.0	3.78	7.0	889.6	26.4	9.92	114.5	0.767	86.1	4.90	149	5.89	0.045	6.61	trace
	48 hour	58.2	27.5	3.59	28.6	893.0	23.8	10.11	114.7	0.847	108.9	4.79	150	5.38	0.024	6.08	none

Hemolysis observed; may falsely elevate AST.

Table S2. Baseline and 48 hour post-MPV complete blood count and biochemistry panel for 20-025.

ID #		WBC (K/ul)	Absolute Neutrophil cells (K/ul)	Absolute Lymphocyte cells (K/ul)	Absolute Monocyte cells (K/ul)	Absolute Eosinophil cells (K/ul)	Absolute Basophil cells (K/ul)	Neutrophil %	Lymphocyte %	Monocyte %	Eosinophil %	Basophil %	RBC (M/ul)	Hemoglobin (g/dL)	Hematocrit %	MCV (fL)	MCH (pg)	MCHC (g/dL)	RDW %	Platelets (K/uL)	MPV (fL)	Presence of clots
20-025	*Baseline*	12.14	3.52	6.56	0.54	1.50	0.01	29.03	54.00	4.48	12.38	0.11	11.73	13.9	44.3	37.8	11.8	31.4	20.9	134	11.7	none
	48 hour	13.64	5.55	7.05	0.37	0.66	0.01	40.66	51.68	2.72	4.84	0.10	10.36	12.6	40.4	39.0	12.2	31.2	20.7	115	11.5	none

ID #		Alanine Transaminase U/L	Aspartate Transaminase U/L	Albumin g/dL	Alkaline Phosphatase U/L	Amylase U/L	Blood Urea Nitrogen mg/dL	Calcium mg/dL	Chloride mmol/L	Creatinine mg/dL	Glucose mg/dL	Potassium mmol/L	Sodium mmol/L	Phosphorus mg/dL	Total Bilirubin mg/dL	Total Protein g/dL	Hemolysis:
20-025	*Baseline*	89.8	34.0	3.75	36.1	2395.6	26.4	9.90	116.7	1.065	69.7	4.10	137	5.41	n.d.	6.54	trace
	48 hour	103.4	33.8	3.75	34.6	2429.6	24.4	10.32	119.1	1.14	90.3	4.75	152	5.59	0.024	6.64	trace

Hemolysis observed; may falsely elevate AST.

n.d. - not detected

Table S3. Baseline and 48 hour post-MPV complete blood count and biochemistry panel for 20-031.

ID #		WBC (K/ul)	Absolute Neutrophil cells (K/ul)	Absolute Lymphocyte cells (K/ul)	Absolute Monocyte cells (K/ul)	Absolute Eosinophil cells (K/ul)	Absolute Basophil cells (K/ul)	Neutrophil %	Lymphocyte %	Monocyte %	Eosinophil %	Basophil %	RBC (M/ul)	Hemoglobin (g/dL)	Hematocrit %	MCV (fL)	MCH (pg)	MCHC (g/dL)	RDW %	Platelets (K/uL)	MPV (fL)	Presence of clots
20-031	*Baseline*	14.26	5.03	5.95	1.04	2.19	0.05	35.30	41.70	7.29	15.33	0.38	12.35	17.2	54.1	43.8	13.9	31.8	20.7	159	12.8	none
	48 hour	12.36	8.84	2.89	0.22	0.39	0.02	71.49	23.38	1.82	3.18	0.13	10.34	13.2	43.4	42.0	12.8	30.4	19.7	148	12.7	none

ID #		Alanine Transaminase U/L	Aspartate Transaminase U/L	Albumin g/dL	Alkaline Phosphatase U/L	Amylase U/L	Blood Urea Nitrogen mg/dL	Calcium mg/dL	Chloride mmol/L	Creatinine mg/dL	Glucose mg/dL	Potassium mmol/L	Sodium mmol/L	Phosphorus mg/dL	Total Bilirubin mg/dL	Total Protein g/dL	Hemolysis:
20-031	*Baseline*	89.6	31.4	4.24	25.9	1394.5	23.2	10.87	118.7	0.788	70.6	5.97	155	6.74	0.022	6.49	none
	48 hour	94.1	41.4	3.99	26.9	1291.3	22.5	10.26	116.5	0.731	92.9	4.61	150	5.63	n.d.	6.07	none

Hemolysis observed; may falsely elevate AST.
n.d. - not detected
